# Acquisition of meiotic DNA repair regulators maintain genome stability in glioblastoma

**DOI:** 10.1038/cddis.2015.75

**Published:** 2015-04-23

**Authors:** M Rivera, Q Wu, P Hamerlik, A B Hjelmeland, S Bao, J N Rich

**Affiliations:** 1 Department of Stem Cell Biology and Regenerative Medicine, Lerner Research Institute, Cleveland Clinic Foundation, Cleveland, OH 44195, USA; 2 Department of Molecular Medicine, Cleveland Clinic Lerner College of Medicine of Case Western Reserve University, Cleveland, OH 44195, USA; 3 Department of Brain Tumor Biology, Danish Cancer Society Research Center, Strandboulevarden 49, Copenhagen DK-2100, Denmark; 4 Department of Cell, Developmental and Integrative Biology, University of Alabama at Birmingham, Birmingham, AL 35294, USA

## Abstract

Glioblastoma (GBM), the most prevalent type of primary intrinsic brain cancer in adults, remains universally fatal despite maximal therapy, including radiotherapy and chemotherapy. Cytotoxic therapy generates double-stranded DNA breaks (DSBs), most commonly repaired by homologous recombination (HR). We hypothesized that cancer cells coopt meiotic repair machinery as DSBs are generated during meiosis and repaired by molecular complexes distinct from genotoxic responses in somatic tissues. Indeed, we found that gliomas express meiotic repair genes and their expression informed poor prognosis. We interrogated the function of disrupted meiotic cDNA1 (DMC1), a homolog of RAD51, the primary recombinase used in mitotic cells to search and recombine with the homologous DNA template. DMC1, whose only known function is as an HR recombinase, was expressed by GBM cells and induced by radiation. Although targeting DMC1 in non-neoplastic cells minimally altered cell growth, DMC1 depletion in GBM cells decreased proliferation, induced activation of CHK1 and expression of p21^CIP1/WAF1^, and increased RPA foci, suggesting increased replication stress. Combining loss of DMC1 with ionizing radiation inhibited activation of DNA damage responses and increased radiosensitivity. Furthermore, loss of DMC1 reduced tumor growth and prolonged survival *in vivo*. Our results suggest that cancers coopt meiotic genes to augment survival under genotoxic stress, offering molecular targets with high therapeutic indices.

Glioblastomas (GBMs) rank among the deadliest of all human cancers, with only modest improvement in patient survival over recent decades. More than 12 000 GBM patients are diagnosed annually in the United States.^1, 2^ Despite aggressive treatment consisting of maximal safe surgical resection, concurrent radiotherapy and chemotherapy, and adjuvant chemotherapy, median survival remains dismal at 12–15 months.^3, 4^ Although numerous molecular targets have been identified in GBM, no molecularly targeted therapy has demonstrated a survival benefit. Radiotherapy remains the cornerstone of post-surgical GBM therapy with modest additional benefit offered by concurrent administration of the oral methylator, temozolomide. However, radioresistance and tumor recurrence is universal in GBM.^4, 5, 6^ Radiation also damages non-neoplastic brain tissue, resulting in cognitive impairment and decreased quality-of-life.^7^ Focal high-dose radiation reduces toxicity to non-neoplastic tissue, but tumor invasion into normal brain regions limits the survival benefit of highly focused radiotherapy techniques, like gamma knife and proton beam, establishing a need for improved combinatorial treatments, such as radiosensitizers.^8, 9^ To date, no radiosensitizer has successfully increased survival with acceptable toxicity in a clinical trial. Based on this background, we sought novel molecular targets that mediate responses to genotoxic stress and have limited function in normal cells.

During mitosis, cells inspect the integrity of their DNA and repair replication errors through cell-state and error-specific mechanisms.^10^ Unrepaired or large regions of DNA damage overwhelm replication mechanisms to induce cell death.^10, 11^ DNA double-strand breaks (DSBs) are detrimental as they cause large-scale chromosomal rearrangements.^10^ The homologous recombination (HR) pathway is primarily used to repair DSBs during S- and G_2_-phases, providing access to both sister and homologous chromosomes as repair templates.^7, 12^ RADiation sensitive 51 (RAD51) is a key recombinase important in HR and replication fork maintenance, functioning in both mitotic and meiotic cells.^7, 12, 13, 14, 15^ Phosphorylated RAD51 replaces replication protein A (RPA) upon DNA loading.^16^ Recombination mediated by RAD51 with the intact DNA template strand results in a relatively error-free repair.^12^

In contrast to mitosis, germ cells undergoing meiosis actively generate genetic diversity through induction of programmed DSBs, which are repaired through HR.^17, 18, 19^ In meiotic HR, RAD51 functions in conjunction with the meiosis-specific recombinase, disrupted meiotic cDNA1 (DMC1). RAD51 and DMC1 are loaded onto DNA by a meiosis-specific accessory protein complex, homologous-pairing protein 2 (HOP2)–meiotic nuclear divisions 1 (MND1), to promote homologous strand invasion and dissociation-loop (D-loop) formation.^20, 21^ D-loops formed using the DMC1–RAD51 complex are more resistant to dissociation as opposed to D-loops formed by RAD51 alone, increasing the likelihood of DNA crossover events.^20^ In addition, DMC1-directed crossovers preferentially utilize the homologous chromosome further increasing genetic variation.^22^

GBM cells commonly harbor genetic lesions that promote unrestrained proliferation but also stimulate genotoxic stress responses. Neoplastic cells do not require perfect fidelity of repair. In fact, dysfunctional repair accelerates genetic evolution of clones, but cancer cells must acquire mechanisms to bypass cell death or senescence in response to exogenous stressors.^11, 23^ Radiotherapy targets proliferating cancer cells by production of reactive oxygen species, leading to generation of DSBs and activation of the DNA damage response (DDR) pathway.^11, 24^ DSBs generated as a result of ionizing radiation (IR) are repaired through HR or non-homologous end joining (NHEJ).^7, 12, 25, 26^ Terminally differentiated neurons are post-mitotic and rely on NHEJ as a means to repair DNA DSBs. Therefore, inhibition of the NHEJ pathway may result in unfavorable normal neural cell toxicity.^26^

The HR pathway is an attractive target as it is linked to increased genetic variation and loss of heterozygosity (LOH).^12, 27^ Multiple HR checkpoints have been proposed as potential therapeutic targets for GBM.^28, 29, 30, 31^ Although the prognostic value of RAD51 expression in GBM is unresolved,^29, 32, 33^ RAD51 is consistently elevated in GBM compared with normal brain.^33^ Reducing RAD51 expression radiosensitizes GBM cells,^29^ but may have a limited therapeutic index because of the potentially toxic effects on non-neoplastic cells. In this study, we investigated the aberrant activity of meiotic HR regulators in glioma, focusing on the meiosis-specific DMC1. Activation of meiotic repair genes in neoplastic cells selectively provides tumor cells with a repair mechanism to evade cell death caused by DNA damage, yet increase genetic diversity to drive clonal evolution.

## Results

### GBM cells express components of the meiotic HR machinery

**Figure 1:**
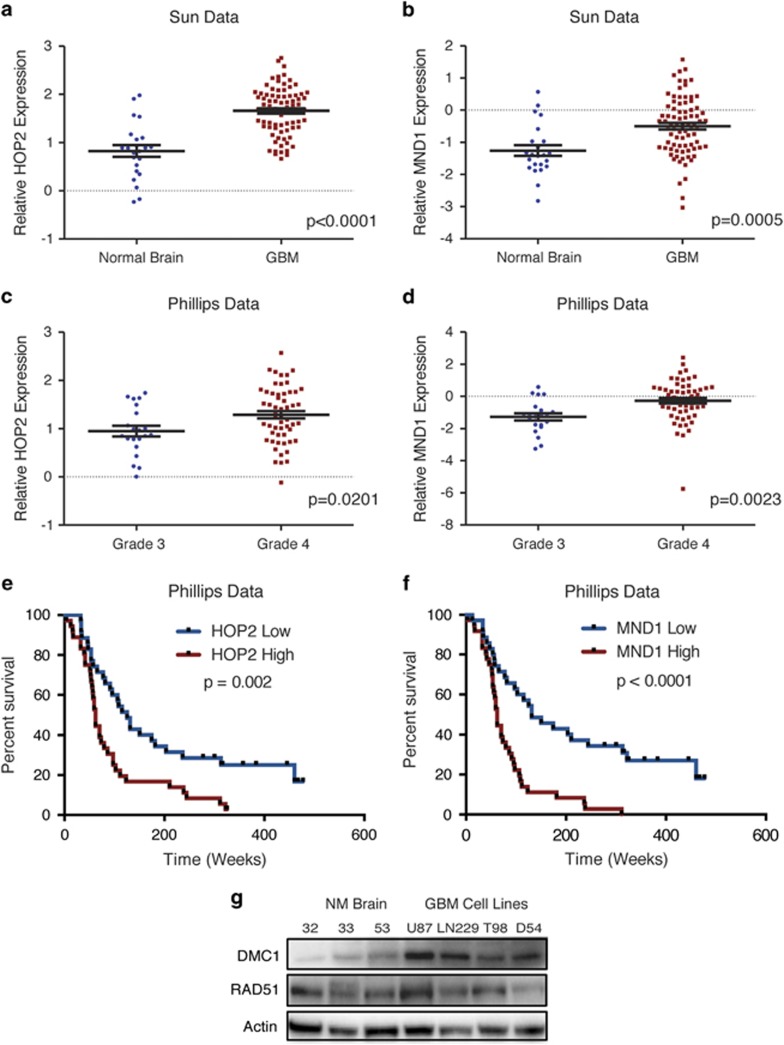
GBM cells express components of the meiotic HR machinery. (**a** and **b**) Oncomine analysis of the Sun database demonstrates elevated (**a**) *HOP2* (*P*=<0.0001) and (**b**) *MND1* (*P*=0.0005) mRNA expression in GBM compared with normal brain (*n*=23 normal brain, *n*=81 GBM; analyzed with unpaired *t*-test). (**c** and **d**) Oncomine analysis of the Phillips database indicates that elevated (**c**) *HOP2* (P=0.0201) and (**d**) *MND1* (P=0.0023) mRNA expression correlates with increased glioma tumor grade (*n*=23 astrocytomas, *n*=76 GBM; analyzed with unpaired *t*-test). (**e** and **f**) Analysis of the Phillips data set available through Oncomine indicates a significant correlation between high (**e**) *HOP2* expression (*n*=35 *HOP2* low, *n*=36 *HOP2* high; *P*=0.002 with log-rank analysis), and (**f**) high *MND1* expression (*n*=35 *MND1* low, *n*=36 *MND1* high; *P*<0.0001 with log-rank analysis) and poor survival. (**g**) DMC1 expression was analyzed by immunoblot analysis in three non-neoplastic brain (NM32, NM33 and NM53) and four GBM cell lines (U87, LN229, T98 and D54)

To interrogate meiosis-related genes in GBM, we initially performed an *in silico* analysis of meiosis-specific HR genes using available annotated glioma expression data sets, including The Cancer Genome Atlas. HOP2–MND1 forms a meiotic complex necessary for loading DMC1 and RAD51 onto single-stranded DNA (ssDNA).^20, 34^ HOP2 and MND1 are more highly expressed in GBM as compared with normal brain (Figures 1a and b) and expression increases with tumor grade (Figures 1c and d). Higher levels of HOP2 or MND1 are both correlated with poor survival (Figures 1e and f), suggesting functional significance in tumors. Although DMC1 mRNA did not inform negative prognosis, likely due to lower variability in expression levels (data not shown), we selected DMC1 for further study as it serves as the downstream effector for the HOP2–MND1 accessory proteins required for the DMC1–RAD51 complex to bind. DMC1 and RAD51 protein levels were analyzed in four GBM cell lines (U87, LN229, T98 and D54) and compared with three neural precursor cultures derived from unaffected white matter in epilepsy resection surgery in adults (NM32, NM33 and NM53) (Figure 1g), as DMC1 is reported to be expressed in normal brain.^35^ RAD51 was expressed at similar levels in both normal and neoplastic brain, befitting its role in somatic cell repair. In contrast, DMC1 protein levels were substantially elevated in GBM cell lines relative to normal brain. These results indicate meiotic HR repair genes are expressed in GBM.

### DMC1 functions in mitotically active glioma cells

**Figure 2:**
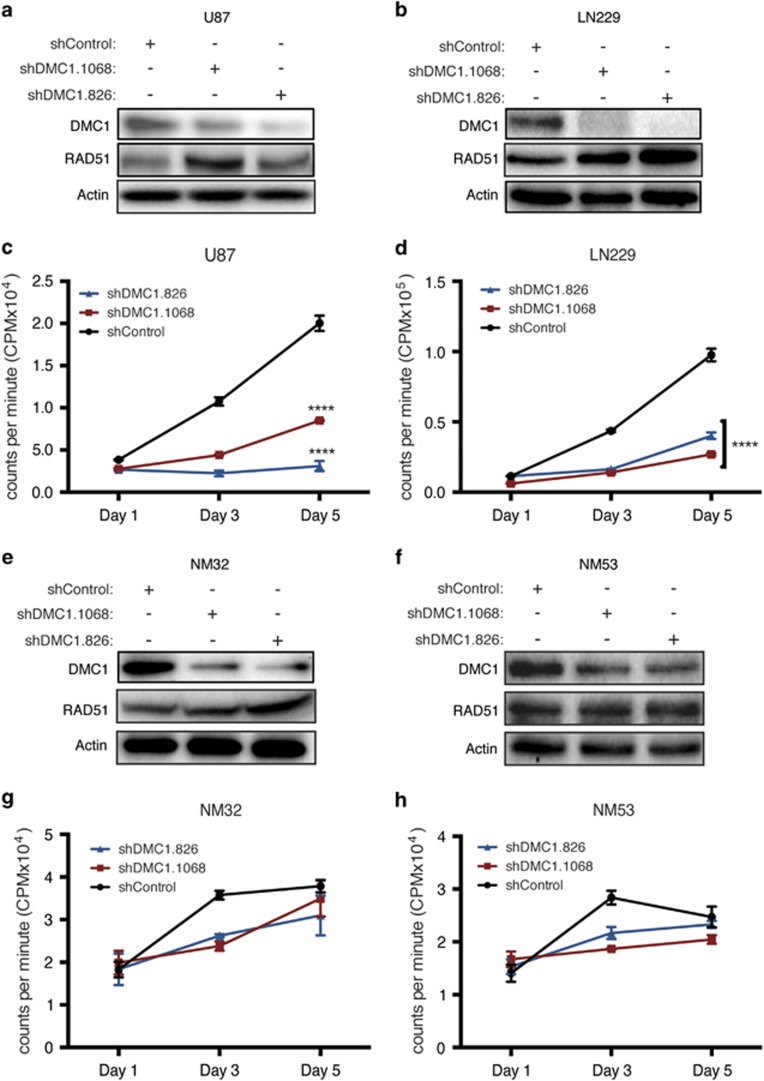
DMC1 depletion inhibits proliferation of GBM cells with minimal effects on non-neoplastic brain cells. (**a** and **b**) U87 (**a**) and LN229 (**b**) cells were transduced with lentivirus expressing either control shRNA (shControl-black) or DMC1-directed shRNA sh1068 (red) and sh826 (blue) and knockdown efficiency was measured by immunoblot analysis. RAD51 protein expression was evaluated in response to DMC1 depletion by immunoblot analysis. Proliferation changes in response to DMC1 depletion was measured in transduced U87 (**c**), LN229 (**d**), by pulsing cells for 4 h with radiolabeled thymidine at the indicated times post-transduction. (**e** and **f**) NM32 (**e**) and NM53 (**f**) cells were transduced with lentivirus expressing either control shRNA (shControl-black) or DMC1-directed shRNA sh1068 (red) and sh826 (blue) and knockdown efficiency was measured by immunoblot analysis. RAD51 protein expression was evaluated in response to DMC1 depletion by immunoblot analysis. Proliferation changes in response to DMC1 depletion was measured in transduced NM32 (**g**) and NM53 (**h**), by pulsing cells for 4 h with radiolabeled thymidine at the indicated time points. *n*=3, errors bars represent S.D.; ****P<0.0001 with ANOVA

To interrogate DMC1 function in mitotically active normal and neoplastic cells, we used two shRNAs targeting non-overlapping sequences of the DMC1 mRNA transcript (shDMC1.1068 and shDMC1.826) relative to a non-targeting shRNA sequence not expressed by mammalian cells (shControl) to control for off-target effects. DMC1 levels were significantly reduced by shRNA transduction in U87 (Figure 2a) and LN229 (Figure 2b) cells. DMC1 and RAD51 share a high sequence homology; to ensure DMC1 shRNA sequences did not target RAD51 in GBM cells, control and DMC1-depleted protein lysates were analyzed for RAD51 expression (Figures 2a and b). Knockdown of DMC1 did not have an effect on RAD51 protein. Although basal DNA synthesis was different between GBM cell lines, DMC1 depletion consistently reduced thymidine incorporation by >50% in U87 (Figure 2c) and LN229 cells (Figure 2d). Although DMC1, but not RAD51, was reduced with DMC1-directed shRNAs, targeting DMC1 in normal brain precursors was slightly less efficient, likely due to lower basal expression levels (*N.B.* immunoblots were overexposed to demonstrate protein levels; Figures 2e and f). In contrast to the results in GBM cells, depletion of DMC1 in non-neoplastic brain cells did not have a significant effect on cell proliferation (Figures 2g and h). Collectively, these results suggest that DMC1 has a unique and functional role in GBM cells, even in the absence of induced damage.

### DMC1 depletion decreases GBM clonogenic survival and cell cycle progression

**Figure 3:**
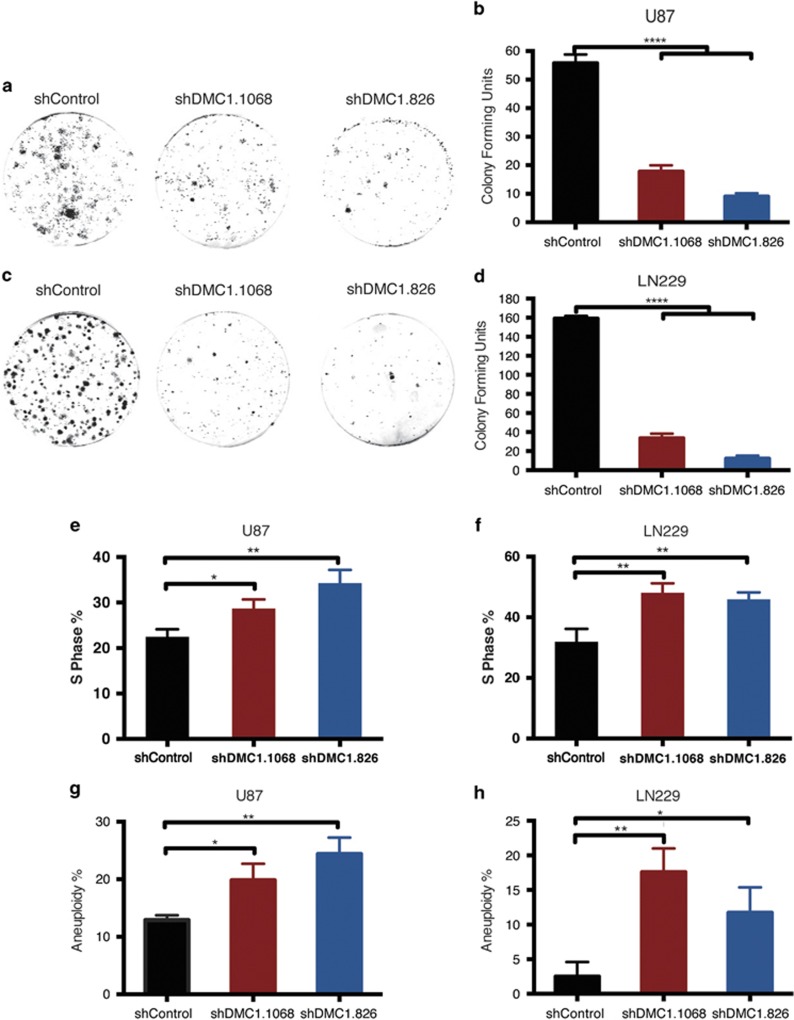
DMC1 depletion reduces clonogenic survival cells and alters the cell cycle profile of GBM cells. (**a**–**d**) DMC1-depleted U87 (**a** and **b**) and LN229 (**c** and **d**) cells were plated at low density and stained with crystal violet for evaluation of clonogenic survival. Representative images (**a** and **c**) are shown and the data quantified (**b** and **d**) by counting the number of colonies formed. (**e**–**h**) DMC1-depleted U87 (**e** and **g**) and LN229 (**f** and **h**) cells were fixed, stained with PI, and cell cycle profiles evaluated using flow cytometric analysis. Percentage of S-phase (**e** and **f**) and aneuploid cells (**g** and **h**) are shown. *n*=3, error bars represent S.D.; NS, no significance; *P<0.05; **P<0.01; ****P<0.0001 with ANOVA

Sustained proliferation and clonogenic survival are hallmarks of neoplastic cells, including GBM. Therefore, we analyzed the effects of DMC1 knockdown on clonogenic survival in U87 and LN229 cells. DMC1-deficient cells were seeded at low density and allowed to grow for 2 weeks. Depleting DMC1 decreased the number of colonies by ≥70% in U87 cells (Figures 3a and b) and ≥75% in LN229 cells (Figures 3c and d) compared with shControl cells. As targeting DMC1 expression reduced both proliferation and colony formation at baseline, we interrogated the effects of DMC1 depletion on cell cycle control in the absence of external genotoxic stressors. Both U87 and LN229 cells displayed an accumulation in S-phase (Figures 3e and f). Aberrant checkpoint control can be associated with development of aneuploidy. In concordance, DMC1 depletion increased the percentage of aneuploidy cells, indicative of increased genomic instability (Figures 3g and h). These data suggest that DMC1 depletion induces cell cycle arrest, increasing endogenous DNA damage.

### Depletion of DMC1 increases replication stress

**Figure 4:**
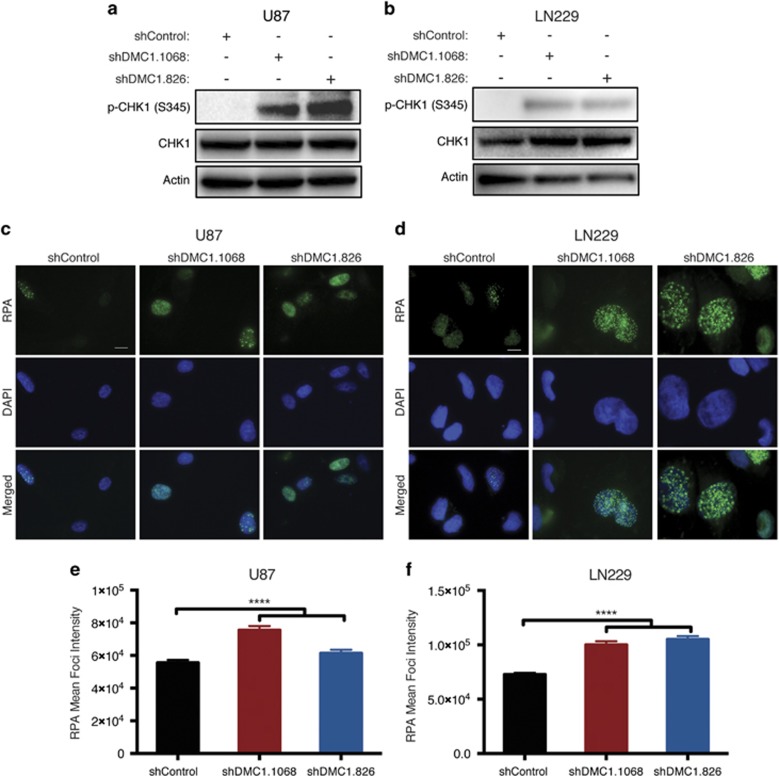
Depletion of DMC1 induces replication stress. (**a** and **b**) Protein expression of phosphorylated CHK1 (S345) and total CHK1 in U87 (**a**) and LN229 (**b**) DMC1-depleted cells was evaluated by immunoblot analysis. (**c** and **d**) Formation of nuclear RPA foci was monitored using immunostaining analysis for RPA (green; nuclei counterstained with DAPI, blue) in U87 (**c**) and LN229 (**d**) DMC1-depleted cells. (**e** and **f**) Quantification of RPA foci in U87 (**e**) and LN229 (**f**) was obtained through ScanR analysis. RPA foci signal was normalized to DAPI per cell. *n*=3, error bars represent S.D.; ****P<0.0001. Scale bar=10 *μ*m

Alterations in the S-phase frequency upon DMC1 targeting are suggestive of accumulating DNA damage with reduced DMC1 expression. To investigate this hypothesis, DMC1-depleted U87 and LN229 cells were analyzed for activation of the checkpoint kinases in the absence of genotoxic stressors. DMC1 depletion induced phosphorylation of checkpoint kinase 1^S345^ (CHK1^S345^), but not checkpoint kinase 2^T68^ (CHK2^T68^) (Figures 4a and b and data not shown), suggestive of increasing replication stress in response to decreased DMC1.

During DNA replication, RAD51 is recruited to ssDNA by formation of RPA-ssDNA filaments. RAD51 then exchanges with RPA to form RAD51-ssDNA nucleoprotein filaments, which inhibit DNA resection by Mre11.^14^ Depleting RAD51 increases accumulation of ssDNA lesions.^14^ Owing to the high sequence homology shared by RAD51 and DMC1, it is plausible that both proteins maintain overlapping functions in mitotic tumor cells. To test this hypothesis, U87 and LN229 DMC1-depleted cells were grown on coverslips and stained for formation of RPA foci. As expected, RPA-positive cells were present in all samples, supporting basal genotoxic stress; however, DMC1 depletion increased the mean intensity of total RPA foci (Figures 4c and f). Persistent RPA foci and phosphorylation of CHK1^S345^ are two markers of replication stress,^36, 37, 38, 39^ both of which are elevated in response to DMC1 depletion. In addition, DMC1-depleted GBM cells have larger nuclei consistent with an increase in aneuploidy (Figures 3g, h, 4c and d), further supporting a critical and specific role of DMC1 in glioma cell responses to genotoxic stress.

### DMC1 depletion radiosensitizes GBM cells

**Figure 5:**
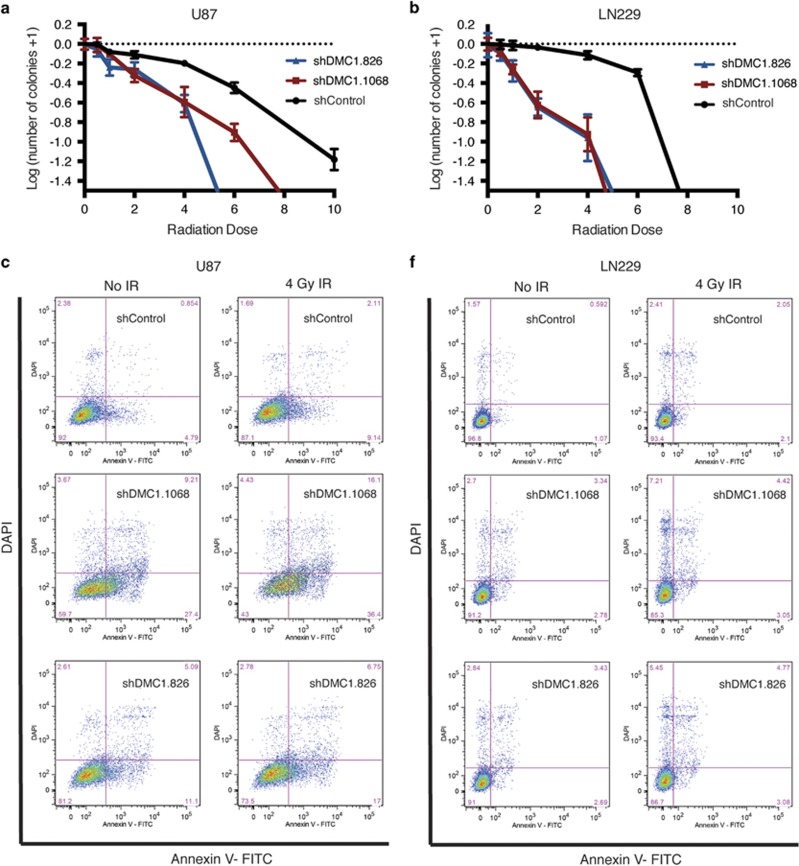

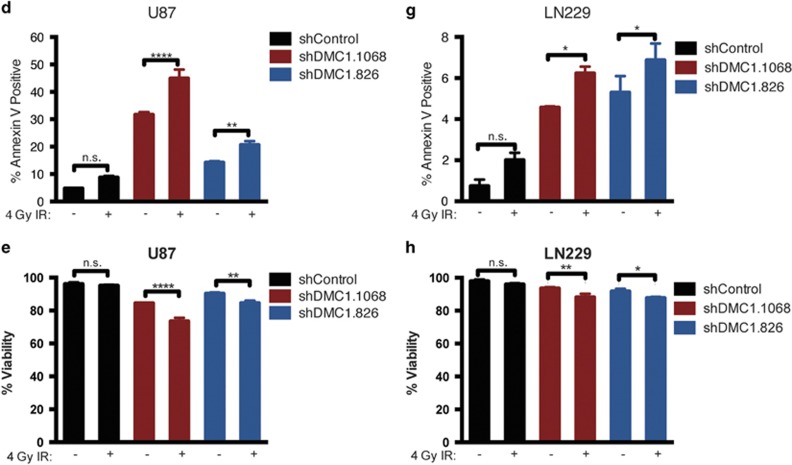
DMC1 depletion radiosensitizes GBM cells to IR. (**a** and **b**) DMC1-depleted U87 (**a**) and LN229 (**b**) cells were plated at low density and evaluated for clonogenic survival in combination with increasing doses of radiation. Colonies were counted for each group and plotted on a log-transformed graph. (**c**–**h**) Combinatorial effects of DMC1 depletion and radiation on apoptosis and viability were evaluated by co-staining with Annexin V-FITC and DAPI, respectively. U87 (**c**) and LN229 (**f**) DMC1-depleted cells were exposed to 4 Gy IR and analyzed by flow cytometry 24 h post-radiation. U87 Annexin V (**d**) and DAPI (**e**) positive cell graphs. LN229 Annexin V (**g**) and DAPI (**h**) positive cell graphs. *n*=3, error bars represent S.D.; NS, no significance; *P<0.05; **P<0.01; ****P<0.0001 with ANOVA

To determine a potential role for DMC1 in response to IR, a form of exogenous genotoxic stress, clonogenic survival was analyzed in DMC1-depleted cells in a dose range of radiation. U87 cells had a 50% survival fraction (EC_50_) at 5 Gy IR in the shControl cells and at 3 Gy IR in DMC1-depleted cells (Figure 5a). LN229 had an EC_50_ at 6 Gy in the shControl cells and at 2 Gy in DMC1-depleted cells (Figure 5b). Combining DMC1 depletion with IR significantly decreased clonogenic survival; however, the observed effects were not entirely through apoptosis. DMC1-depleted U87 and LN229 cells were exposed to 4 Gy IR and early apoptosis was quantified via Annexin V staining. In the absence of IR, decreasing DMC1 in U87 cells increased Annexin V staining from a baseline of ~5% for shControl to ~35% and 16% for shDMC1.1068 and shDMC1.826, respectively (Figures 5c and d). Exposure to 4 Gy IR had a modest apoptotic effect on shControl cells (baseline 5% apoptosis without IR to ~10% apoptotic fraction 24 h post-IR), but Annexin V staining significantly increased from 35% to 50% for shDMC1.1068 cells and from 16% to 25% for shDMC1.826 cells (Figures 5c and d). DMC1 depletion also increased sensitivity to IR in LN229 cells, albeit with a more modest effect. Twenty-four hours post-IR (4 Gy), shControl cells had a minimal increase in Annexin V staining (from 2 to 4% Figures 5e and f). Annexin V staining increased from 4 to 6% for shDMC1.1068 and from 5 to 8% for shDMC1.826 (Figures 5e and f). Viability was quantified by exclusion of DAPI staining. U87 cell viability was high (97%) in shControl cells, but significantly decreased in shDMC1.1068 (84%) and shDMC1.826 (90%) cells in the absence of IR. Treatment with 4 Gy IR significantly decreased the percentage of viable cells in shDMC1.1068 (75%) and shDMC1.826 (84%) cells, with a minimal effect on shControl cells (96%) (Figures 5c and d). A similar effect was observed in LN229 cells. DMC1 depletion alone decreased the percentage of viable cells in shDMC1.1068 (84%) and shDMC1.826 (92%) cells compared with the shControl cells (97%). Treatment with 4 Gy IR had a more pronounced effect on shDMC1.1068 (88%) and shDMC1.826 (87%) cells compared with the shControl cells (96%) (Figures 5c and d). Collectively, these data indicate that DMC1 depletion increases sensitivity to IR.

### Depletion of DMC1 inhibits radiation-induced activation of the DDR

**Figure 6:**
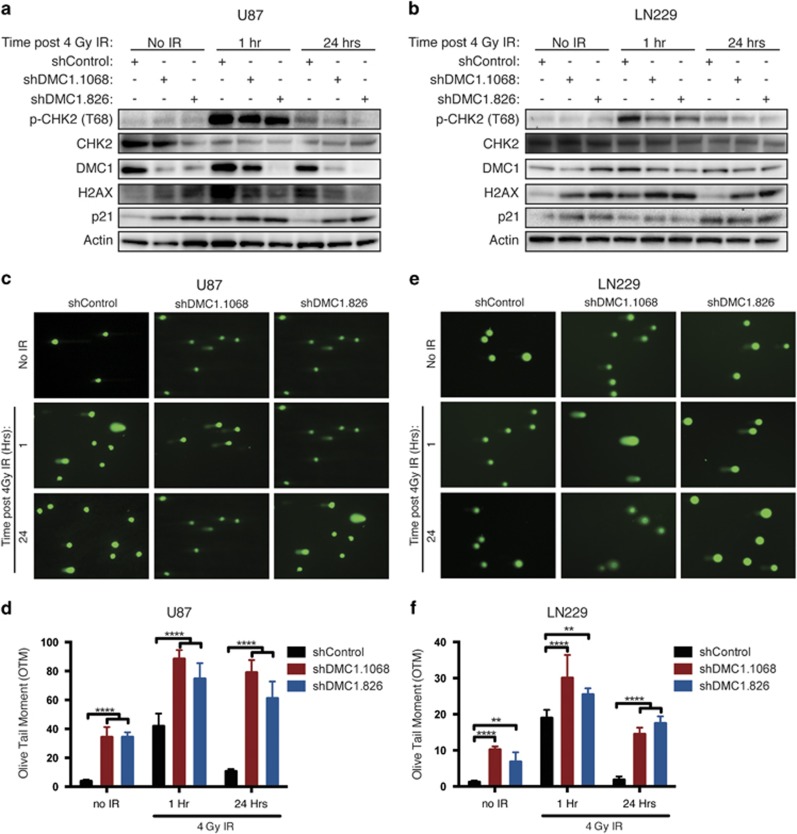
DMC1 depletion attenuates activation of the DDR. (**a** and **b**) Activation of the DDR was assessed by immunoblot analysis of phosphorylated CHK2 (T68), total CHK2, DMC1, p21^CIP1/WAF1^ and *γ*-H2AX in U87 (**a**) and LN229 (**b**) DMC1-depleted cells exposed to 4 Gy radiation and harvested at the indicated time points. (**c**–**f**) DNA damage of baseline and radiation-treated DMC1-depleted cells was evaluated at the indicated time points following 4 Gy radiation with the alkaline comet assay in U87 (**c** and **d**) and LN229 (**e** and **f**) cells. Representative images (**c** and **e**) are shown. Quantification of comet tails for U87 (**d**) and LN229 (**f**) was done using the Casplab software and is represented as OTM. *n*=3, error bars represent S.D.; **P<0.01; ****P<0.0001 with two-way ANOVA

Radiation sensitivity induced by DMC1 depletion supports our hypothesis that DMC1 is a regulator of GBM genomic stability. Endogenous and IR-induced DNA DSBs are primarily repaired through HR; therefore, we expected an increase in DNA damage as a result of DMC1 depletion. Analysis of DMC1 levels in response to IR resulted in increased DMC1 protein expression in U87 and LN229 cells as early as 1 h following IR (Figures 6a and b), indicating DMC1 may be involved in repairing DNA DSBs induced by radiation. Evaluation of the DDR pathway in U87 and LN229 cells transduced with shDMC1 demonstrated that targeting DMC1 induced basal expression of the cyclin-dependent kinase inhibitor, p21^CIP1/WAF1^, and *γ*-H2AX, a marker of DNA damage (Figures 6a and b). Activation of CHK1^S345^, but not CHK2^T68^, effector kinase was confirmed with DMC1 knockdown (Figures 4a, b, 6a and b). IR independently increased *γ*-H2AX, p21^CIP1/WAF1^, and phosphorylation of CHK2^T68^. Notably, CHK2^T68^ activation was greater in shControl cells compared with DMC1-depleted cells, but CHK2^T68^ activation significantly resolved 24 h post-IR, despite sustained elevation of p21^CIP1/WAF1^ and *γ*-H2AX (Figures 6a and b). These data indicate DMC1-depleted cells do not effectively sense IR-induced DNA damage or activation of downstream effector proteins is repressed.

The elevated expression of *γ*-H2AX in the absence of external stressors indicates DMC1 depletion alone leads to increased DNA damage. An alkaline comet assay was used to evaluate the extent of DNA damage at baseline and post-IR in shControl and DMC1-depleted cells. DNA damage was quantified by olive tail moment (OTM).^40^ The baseline DNA damage in U87 shControl cells was minimal (3.8 OTM) compared with damage in cells transduced with either shDMC1.1068 or shDMC1.826 (34.2 and 34.3 OTM, respectively; Figures 6c and d). Similarly, the baseline damage in LN229 shControl cells was minimal (1.2 OTM) compared with cells expressing shDMC1.106 and shDMC1.826 (10.2 and 6.9 OTM, respectively; Figures 6e and f). One-hour post-IR, U87 shControl cells displayed a significant increase in damage (41.9 OTM); however, DMC1 depletion induced much greater effects in combination with IR (88.4 and 74.6 OTM in shDMC.1068 and shDMC1.826, respectively). LN229 shControl cells also displayed a significant increase in DNA damage (18.9 OTM) 1 h following 4 Gy IR that was exacerbated by DMC1 depletion (30 and 25.5 OTM in shDMC.1068 and shDMC1.826, respectively). The acquired DNA damage was largely repaired in both U87 and LN229 shControl cells by 24 h (10.6 and 1.8 OTM, respectively). Cells transduced with shDMC1.1068 and shDMC1.826 maintained elevated levels of damage (79 and 61 OTM in U87 and 14.5 and 17.5 OTM in LN229, respectively; Figures 6c–f). DNA damage in DMC1-depleted cells was greater than the baseline level 24 h after 4 Gy IR. Taken together, these results suggest depletion of DMC1 increases endogenous DNA damage and impairs resolution of IR-induced DSBs in GBM cells.

### Depletion of DMC1 attenuates *in vivo* tumor growth and increases survival of tumor-bearing animals

**Figure 7:**
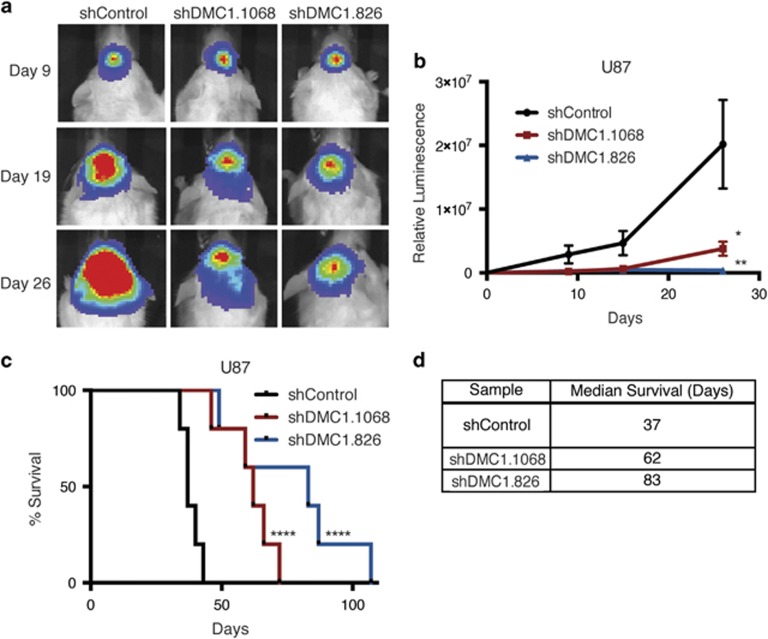
Depletion of DMC1 attenuates tumor growth *in vivo*. (**a** and **b**) GFP-Luciferase expressing U87 cells transduced with control shRNA or DMC1-directed shRNA were implanted intracranially in NSG mice, five mice per group (**a**) Mice were imaged *in vivo* using the IVIS Lumina at the indicated days post-implantation. (**b**) Relative luminescence values for all three groups were plotted and analyzed over time. Errors bars represent S.E.M.; *P<0.05; **P<0.01 with ANOVA. (**c**) Mice were killed at first sign of neurological deficit and Kaplan–Meier survival curves plotted. ****P<0.0001 with log-rank test. (**d**) Table with medial survival in days for each experimental arm

Our collective *in vitro* data suggest that DMC1 contributes to the maintenance of genomic stability in GBM cells. To evaluate the effects of DMC1 depletion *in vivo,* GFP-luciferase expressing U87 cells were transduced with shControl or one of two DMC1 shRNAs then implanted intracranially in the frontal lobes of immunocompromised mice. Tumor growth was measured *in vivo* using bioluminescence. Nine days after implantation, all three groups had tumors of similar size. By day 26, the shControl arm had significantly larger tumors compared with the shDMC1.1068 or shDMC1.826 arms (Figures 7a and b). Targeting DMC1 significantly extended the lifespan of tumor-bearing hosts relative to control animals, with a median survival of 37 days in the shControl arm and 62 and 83 days in the shDMC1.1068 and shDMC1.826 arms, respectively (Figures 7c and d). As the NOD scid gamma (NSG; NOD.Cg-Prkdc^scid^ Il2rg^tm1Wjl^/SzJ) mouse model is highly radiosensitive with 100% death of hosts at therapeutic radiation doses, comparative studies with addition of IR were not possible.

## Discussion

The genomic landscape of GBM has revealed numerous molecular targets that have informed new models of gliomagenesis and therapeutic resistance, but these molecular findings have not impacted the clinical outcome of patients. Targeted therapeutics used as monotherapies have not extended overall survival for GBM patients, leading to the interrogation of combinatorial therapies to enhance the effects of radiotherapy and chemotherapy, but these studies have been largely disappointing, including bevacizumab as an adjuvant agent in newly diagnosed patients undergoing chemoradiation.^41, 42^ Bevacizumab has been the most active targeted therapeutic for GBM as a monotherapy but fails to increase overall survival for newly diagnosed or recurrent patients, and adds significant toxicity. To discover novel therapeutic targets, we used an analysis of genes that function only in meiosis with the rationale that normal somatic cells do not generally express these genes and that cancers may coopt meiotic gene function to survive stress. In this study, we investigated the therapeutic potential of targeting the meiotic recombinase, DMC1, in GBM cells.

Our approach to elucidating the role of DMC1 in GBM cells was selected based on its enzymatic function in meiotic DNA repair. DMC1 recombination requires hydrolysis of ATP,^43^ a feature that can be exploited for development of small molecule inhibitors. Interrogation of DMC1 function revealed a level of redundancy with known RAD51 activities. RAD51 maintains replication fork integrity by binding to RPA-coated ssDNA; therefore, it is likely DMC1 also maintains replication fork integrity in GBM cells. Based on our data, DMC1 depletion induced a baseline increase in RPA foci, phosphorylation of CHK1^S345^, and accumulation of cells in S-phase. Collectively, these data support a role of DMC1 in replication fork integrity.

In addition, DMC1—either alone or in a complex with RAD51—is potentially utilized to repair DNA through HR. DMC1-depleted cells display a significant amount of accumulated DNA damage and decreased resolution of IR-induced damage. Of note, DMC1 depletion significantly attenuated DDR response to IR. The attenuation of the DDR pathway may result from saturation of CHK1 phosphorylation, thereby overwhelming the DDR machinery to fully activate CHK2. Our data, however, are not conclusive as to which process, replication fork maintenance or repair through HR, is the major role of DMC1.

DMC1 potentially has other unexplored functions that provide a survival advantage to GBM cells. Recent studies evaluating meiotic genes, including DMC1, found expression of these genes in melanoma, lymphoma, cervical, breast and colon cancers.^44, 45, 46^ Expression of DMC1 was previously shown to increase in response to IR in various cancer cell types, with a proposed role of decreasing ploidy in cells that have undergone mitotic catastrophe.^45^ Although non-neoplastic aneuploidy cells would usually undergo apoptosis, cancer cells evade cell death and utilize survival mechanisms, potentially involving DMC1, to return to a sustainable ploidy state.

Along with altered chromosome number and aberrations, the complexity of cancer cells is difficult to appreciate by cytological analysis alone, as uniparental isodisomy may not be detected. Heterozygous polymorphisms that are usually silent can be lost during HR and template switching, thereby supporting the emergence of cancer cell sustaining mutations.^27, 47^ The preferential use of the homologous chromosome by DMC1 during meiotic recombination may be preserved in GBM cells, leading to copy number neutral LOH and/or chromosomal duplications, thus increasing *inter*- and *intra*-tumoral heterogeneity.

Although limitations in radiotolerance of the immunocompromised mouse model used in our studies prevented *in vivo* studies of combined DMC1 depletion and IR, we found delayed tumor progression and increased survival with DMC1 depletion supports the therapeutic potential of DMC1 targeting. Our *in vitro* combination studies demonstrated a sensitization of DMC1-depleted cells to IR with only a modest increase in apoptosis; therefore, drugs targeting other survival pathways may enhance the benefit of targeting DMC1, establishing a synthetically lethal combination.

The concept of synthetic lethality has been eloquently demonstrated with poly ADP-ribose polymerase (PARP) inhibitors and their selectivity for HR-deficient cells, particularly those with mutations in BRCA1 and BRCA2.^48, 49^ PARP is required for detection and signaling of single-strand break repair (SSBR).^23^ The efficacy of PARP inhibitors as a cytotoxic agent increases 1000-fold in HR-defective cancer cells.^49^ The proposed hypothesis is that while a defect in one DNA repair pathway is sustainable, inhibition of multiple pathways prevent compensation of repair and therefore is genomically unsustainable.^23, 49^

PARP, topoisomerase, and other DDR inhibitors are currently under clinical investigation to determine their safety and efficacy in patients with GBM.^11, 23, 50^ These studies can be expanded to include DMC1 as an inhibitor of HR. Our results show a marked defect in DDR activation with DMC1 depletion and combination of a DMC1 inhibitor with treatment modalities that target various DNA repair pathways could effectively target GBM cells.

Advances in GBM treatment have only provided minimal therapeutic effect. Our work underscores the complexity of this disease and the exigency to explore previously overlooked mechanisms of treatment resistance and evasion of apoptosis. Our studies are the first to establish a functional role, beyond expression, of DMC1 in cancer cells. In addition, we present a novel role in replication progression for DMC1 in either meiotic or mitotic cells. Importantly, we demonstrate the high therapeutic potential for targeting DMC1 because of the minimal effect depletion of DMC1 had on non-neoplastic brain cells. Acquisition of meiotic regulators provides a cellular advantage that we can exploit to develop cancer-specific treatments with minimal toxicity.

## Materials and Methods

### Cell lines, culture and irradiation

The human GBM cell lines U87MG, T98 and LN229 were acquired from American Type Culture Collection (ATCC, Manassas, VA, USA). D54MG is the Duke University subline of A-172. U87MG, T98, LN229 and D54MG glioma cells were maintained in Dulbecco's modified Eagle's medium (DMEM; Gibco, Grand Island, NY, USA) supplemented with 10% fetal bovine serum (Gibco). All cells were cultured at 37 °C in an atmosphere of 5% CO_2_. For cell counting before each experiment, a single-cell suspension was achieved using TrypLE (Invitrogen, Grand Island, NY, USA). Cells were irradiated in a JL Shepherd Mark I ^137^Cs irradiator (San Fernando, CA, USA).

### Immunoblot analysis

Immunoblot analyses were performed as previously described.^51, 52^ Briefly, RIPA protein extracts were separated by 4–12% Novex NuPAGE SDS-PAGE Gel System (Invitrogen) and transferred to nitrocellulose membranes (Advantech, Dublin, CA, USA) using the Trans-Blot Cell System (Bio-Rad, Hercules, CA, USA). The membranes were blocked with 5% (wt/vol) dry milk in PBS-Tween-20 (0.5% vol/vol) and probed with primary antibodies against p-CHK2 (T68), p-CHK1 (S345), CHK2, CHK1 and p21^CIP1/WAF1^ (1 : 1000; CST, Beverly, MA, USA), DMC1 and RAD51 (1 : 1000; SCBT SC-8349, Santa Cruz, CA, USA), *γ*-H2AX (1 : 2000; Millipore, Temecula, CA, USA) or *β*-actin (1 : 5000; Sigma-Aldrich, St. Louis, MO, USA) as a loading control. ECL detection system was used according to the manufacturer's instructions (GE Healthcare, Little Chalfont, UK).

### Alkaline comet assay

DNA damage repair was assessed by single-cell electrophoresis assay under alkaline conditions as previously described^53^ (Trevigen, Gaithersburg, MD, USA). Briefly, cells were resuspended to 1 × 10^5^ cells/ml, mixed with agarose (1 : 10) and plated on comet slides. Cells were lysed and placed in alkali unwinding solution. After electrophoresis, DNA was stained with sybr gold DNA stain (Invitrogen). Images were taken using a Leica DM4000 Upright microscopy (Leica, Buffalo Grove, IL, USA). Analysis was done using CASPLab software (CASPLab.com).^40^

### Cell cycle analysis

In all, 250 000 cells were plated overnight in DMEM supplemented with 10% fetal bovine serum in a 10-cm tissue culture plate, and harvested 24 h later. For cell cycle analysis, floating and adherent cells were collected and fixed with 70% EtOH, stained with propidium iodide and analyzed using the FACScan and LSRII (BD Bioscience, San Diego, CA, USA). Data were analyzed using ModFit LT software (Verity Software House, Topsham, ME, USA).

### Colony formation assay

Colony formation assays were performed as previously described.^53^ Briefly, GBM cells were plated in triplicate and were unirradiated or irradiated with indicated doses in DMEM, 10% FBS (5000 cells per well). Plates were processed 2 weeks later. Cell colonies were fixed with methanol and stained with a 0.5% crystal violet solution. Plates were imaged using plate-scanning software on an inverted microscope (Leica, Wetzlar, Germany). An area threshold of 500 pixels (equivalent to a colony of ~50 cells) was set and a total area positive for colony formation was calculated and counted for each plate.

### Thymidine incorporation assay

Thymidine incorporation into the DNA was measured with a scintillation counter as previously described.^51, 52^ Briefly, 10 000 cells per well were plated in a 12-well tissue culture plate. Cells were allowed to grow for 24 h, and then labeled for 4 h with 4 *μ*Ci [^3^H]- thymidine, fixed in 10% trichloroacetic acid and lysed in 0.2 N NaOH at the indicated time points.

### Immunofluorescence imaging

Detection of total RPA (1 : 250; Millipore) and was performed as described previously.^36^ GBM cells were grown on coverslips and fixed 24 h after plating. Cells were then immunostained for RPA. Secondary detection was accomplished using Alexa Fluor 488 goat anti-mouse IgG (Invitrogen). Nuclei were counterstained with DAPI. Images were taken using a Leica DM4000 Upright microscopy (Leica). Slides were scanned with the ScanR screening station and data acquired analyzed using ScanR analysis software (Olympus, Hamburg, Germany). Signal was normalized to DAPI per cell.

### Cell viability and apoptosis assays

To measure the differential sensitivity of DMC1 depletion to radiation U87 and LN229 DMC1-depleted GBM cells (100 000 cells per well of a six-well plate, plated in triplicate) were unirradiated or exposed to 4 Gy radiation in DMEM, 10% FBS, 24 h after plating. Viability and apoptosis was measured 24 h post-radiation. For viability, cells were stained with DAPI, and for apoptosis cells were co-stained for Annexin V-FITC (BD Bioscience). Stained cells were analyzed using BD LSRFortessa cell analyzer (BD Bioscience).

### Lentiviral shRNA production and transduction

HEK 293T cells were acquired from ATCC. Cells were plated at density of 2 million cells per 10 cm plate and allowed to grow overnight. Calcium phosphate was used to transfect DNA (Clontech, Mountain View, CA, USA) and viral media collected 48 h later. Virus was concentrated using PEG-It virus precipitation solution (SBI, Mountain View, CA, USA), aliqouted and stored at −80 °C. Puromycin (Fisher, Pittsburgh, PA, USA) was used for selection of transduced cells.

### Animals and *in vivo* studies

All animal studies described were approved by the Cleveland Clinic Foundation Institutional Animal Care and Use Committee and conducted in accordance with the NIH Guide for the Care and Use of Laboratory Animals. For intracranial implantation studies, GFP-Luciferase-shRNA (control or shDMC1) expressing U87 cells were implanted into the right frontal lobes of immunocompromised NSG (NOD.Cg-Prkdc^scid^ Il2rg^tm1Wjl^/SzJ) mice (Charles River Laboratories, Wilmington, MA, USA). Tumor growth was monitored by intraperitoneal injection of 50 *μ*l 30 mg/ml luciferin and analyzed using the *In Vivo* Imaging System (IVIS, Perkin Elmer, Santa Clara, CA, USA) three times per week. Mice were monitored daily for neurological impairment at which time they were killed and brains were removed to evaluate for tumor development.

### Retrospective analysis of *HOP2* and *MND1* gene expression in human gliomas

Correlations between glioma expression compared with normal brain, glioma grade, and patient survival and *HOP2–MND1* gene expression levels were determined through analysis of Sun and Phillips brain data sets, respectively, which are available through Oncomine (Compendia Biosciences, http://www.oncomine.org/). High and low groups were defined as above and below the mean, respectively.

### Statistical analysis

Statistical significance was calculated with GraphPad Prism Software utilizing a one-way or two-way ANOVA with a Bonferroni's *post hoc* test, Student's *t*-test, or log-rank (Mantel–Cox) test, where appropriate (GraphPad Software Inc., San Diego, CA, USA). Data are represented as the mean±S.D.

## Abbreviations

GBM: glioblastoma
DSB: double-strand breaks
HR: homologous recombination
DMC1: disrupted meiotic cDNA1
RAD51: RADiation sensitive 51
CHK1: checkpoint kinase 1
CHK2: checkpoint kinase 2
RPA: replication protein A
IR: ionizing radiation
HOP2: homologous-pairing protein 2
MND1: meiotic nuclear divisions 1
D-Loop: dissociation-loop
DDR: DNA damage response
NHEJ: non-homologous end joining
ssDNA: single-stranded DNA
LOH: loss of heterozygosity
PARP: poly ADP-ribose polymerase
SSBR: single-strand break repair
OTM: olive tail moment
NSG: NOD scid gamma mouse
